# Advances in the Molecular and Cellular Biology of *Strongyloides* spp.

**DOI:** 10.1007/s40475-019-00186-x

**Published:** 2019-09-13

**Authors:** Tegegn G. Jaleta, James B. Lok

**Affiliations:** 1 Department of Pathobiology, School of Veterinary Medicine, University of Pennsylvania, Philadelphia, PA, USA

**Keywords:** *Strongyloides*, Genome, Transcriptome, Proteome, Transgenesis, CRISPR/Cas9

## Abstract

**Purpose of Review:**

This paper constitutes an update of recent studies on the general biology, molecular genetics, and cellular biology of *Strongyloides* spp. and related parasitic nematodes.

**Recent Findings:**

Increasingly, human strongyloidiasis is considered the most neglected of neglected tropical diseases. Despite this, the last 5 years has seen remarkable advances in the molecular biology of *Strongyloides* spp. Genome sequences for *S. stercoralis, S. ratti, S. venezuelensis*, *S. papillosus*, and the related parasite *Parastrongyloides trichosuri* were created, annotated, and analyzed. These genomic resources, along with a practical transgenesis platform for *Strongyloides* spp., aided a major achievement, the advent of targeted mutagenesis via CRISPR/Cas9 in *S. stercoralis* and *S. ratti*. The genome sequences have also enabled significant molecular epidemiologic and phylogenetic findings on human strongyloidiasis, including the first genetic evidence of zoonotic transmission of *S. stercoralis* between dogs and humans. Studies of molecular signaling pathways identified the nuclear receptor *Ss*-DAF-12 as one that can be manipulated in the parasite by exogenous application of its steroid ligands. The chemotherapeutic implications of this were unscored by a study in which a *Ss*-DAF-12 ligand suppressed autoinfection by *S. stercoralis* in a new murine model of human strongyloidiasis.

**Summary:**

Seminal advances in genomics of *Strongyloides* spp. have transformed research into strongyloidiasis, facilitating fundamental phylogenetic and epidemiologic studies and aiding the deployment of CRISPR/Cas9 gene disruption and editing as functional genomic tools in *Strongyloides* spp. Studies of *Ss*-DAF-12 signaling in *S. stercoralis* demonstrated the potential of this pathway as a novel chemotherapeutic target in parasitic nematodes.

## Introduction

Strongyloidiasis, caused mainly by *Strongyloides stercoralis*, is one of the most neglected soil-borne tropical diseases. Human infections with *Strongyloides fuelleborni* and *S. fuelleborni kellyi* have also been reported in Asia, Papua New Guinea, and some African locales [1–5]. Globally, *S. stercoralis* is reported to infect more than 370 million people mainly in tropical and subtropical regions [6]. However, this number may be a significant underestimate due to insensitive diagnostic techniques [6, 7]. Strongyloidiasis is generally regarded as a problem in underdeveloped nations, but this disease is also endemic in economically stressed and institutionalized populations in developed countries [7]. Travelers, tourists, military personnel, and immigrants from endemic regions can spread the parasite in developed countries [7]. *S. stercoralis* has a cosmopolitan distribution in tropical and subtropical regions of the world with the prevalence ranging from 5 to 40%, with higher prevalence among young individuals and socioeconomically marginalized communities [7]. The high prevalence of *S. stercoralis* in tropical and subtropical regions is mainly attributed to high temperature, high moisture, poor sanitation, poor hygiene, and occupations such as farming and mining that increase the chance of individuals coming in contact with soils contaminated with infective third-stage larvae (iL3) of *S. stercoralis* [7]. In the USA, a series of small studies in selected populations have shown that between 0 and 6.1% of persons sampled were infected. However, studies of immigrant populations in the USA have revealed prevalence of infection as high as 49.2% [7, 8].

*S. stercoralis* infections have also been described in dogs, cats, and several nonhuman primates [2, 9–12]. The prevalence of *S. stercoralis* in dogs ranges from 0 to over 45%, with younger dogs and puppies more likely to be infected and to exhibit serious or fatal illness [2]. *S. stercoralis* iL3s in the external environment generally infect the host by skin penetration (percutaneous route) [1], but in addition, transmammary transmission has been observed in lactating bitches [13, 14]. The transmammary route of transmission of *S. stercoralis* has not been reported in humans thus far. Natural infections of dogs with a human strain of *S. stercoralis* have been reported in rural communities in Southeast Asia [15••, 16, 17]. Hence, the zoonotic importance and the potential of dogs as a reservoir of *S. stercoralis* to humans are receiving attention in the scientific community [15••, 17].

In immunocompetent and healthy individuals, *S. stercoralis* causes chronic asymptomatic infections with few or no symptoms or lesions [18]. Symptomatic individuals with chronic strongyloidiasis may present with gastrointestinal indicators such as diarrhea, constipation, and intermittent vomiting [19]. Cutaneous lesions such as urticaria and rashes are also common in symptomatic chronic strongyloidiasis [19]. However, in immunocompromised patients, strongyloidiasis can cause severe generalized and complicated fatal systemic infections from exponential multiplication and dissemination of autoinfective *S. stercoralis* third-stage larvae (aL3) which involve gastrointestinal, respiratory, central nervous systems [20, 21]. Hyperinfection syndrome occurs when aL3 penetrate the small intestinal mucosa in large numbers and migrate somatically, resulting in severe systemic symptoms and organ failure [22]. Hyperinfection syndrome is commonly reported in individuals under corticosteroid treatment and cases of human T-lymphotropic virus type 1 (HTLV-1) co-infection with *S. stercoralis* [21, 23]. Complicated strongyloidiasis due to hyperinfection and dissemination of autoinfective larvae into vital organs such as liver, lung, and brain can result in death in 85% of the cases [24] unless anti-*Strongyloides* chemotherapy is given in time [25, 26]. Migrating autoinfective larvae also disseminate enteric microorganisms to these organs, which requires administration of broad spectrum antibiotics to prevent fatal septic shock and meningitis [27].

A previous review in *Current Tropical Medicine Reports* [28] covered contemporary advances in the cellular and molecular biology of *Strongyloides* spp. up to 2014. The present review is intended to provide updates in the general areas covered in that review.

## Basic Biology

### Life Cycle

*Strongyloides* spp. have unique and complex life cycles that alternate between parasitic and free-living generations [29]. The host is infected when iL3 penetrate the host skin, migrate through the host body, and finally establish themselves in the mucosa of small intestine. During this process, the iL3 molt twice to develop to parasitic adult females. From more than 50 species of the genus *Strongyloides* described, there is not a single report of parasitic males. The adult parasitic female spends its life embedded in the mucosa of the small intestine mainly in the duodenal region. The parasitic female produces eggs by mitotic parthenogenesis (asexual reproduction). *S. stercoralis* eggs hatch in the small intestine and male and female first-stage larvae (L1) pass to the environment with the host feces. A proportion of female L1 in the extrinsic environment develops via a homogonic cycle directly to iL3, which must infect a susceptible host in order to develop further. The remainder of the L1s develops via a heterogonic cycle to free-living males and females, undergoing four molts in the process. Free-living adults reproduce sexually to produce only female progeny that develop to iL3 that require a host for further development [30, 31]. Free-living development by most *Strongyloides* spp. is limited to a single generation, but *Strongyloides planiceps*, a parasite of cats, can undergo up to nine sequential generations of free-living development, but with decreasing brood sizes in each [32]. iL3 of *S. stercoralis* can live in the environment (soil) for about 2 weeks until they encounter a suitable host, where they penetrate the host skin to initiate the parasitic life cycle. As discussed above, *S. stercoralis* also has the unique ability to execute autoinfection when female L1 develop precociously to autoinfective aL3 in the intestine instead of passing with the host feces as L1. These aL3 penetrate the intestinal wall or the perianal skin to continue re-infecting the same host through repeated generations. In immunocompetent patients, well-regulated autoinfective cycling of *S. stercoralis* can maintain chronic subclinical infection for decades without further input if iL3 from the environment [33]. By contrast, in immunocompromised individuals, the autoinfective cycle proceeds in unregulated fashion and can result in hyperinfection syndrome with geometric increases in parasite burden, which may result in fatal illness from dissemination of autoinfective larvae and pathogenic gut microbes. Due to its unique and complicated life cycle, *S. stercoralis* poses a serious risk to public health in both endemic and nonendemic regions, unless appropriate health and environmental management strategies are properly and effectively implemented.

### Germline Organization, Reproduction, and Sex Determination

The model nematode, *Caenorhabditis elegans*, has a tubular gonad in both sexes [34]. In hermaphrodites, the gonad has two arms, one extending posteriorly and the other anteriorly with both arms ending at vulva. In males, the gonad has only one arm with a caudal opening. The distal tip cell sits at the terminus of each gonadal arm and signals the nearby germ cells to proliferate mitotically [35]. Germ cells exit the mitotic cycle when they move away from the distal cell signal. At this point, they initiate meiosis and begin to differentiate into gametes. Most nematodes, including *C. elegans* and *Pristionchus pacificus*, maintain their stem cell populations at the distal end of each gonadal arm ensuring a constant flow of differentiated germ cells [36]. The gonad of the *C. elegans* hermaphrodite is packed with mitotically dividing germ cells, crescent-shaped nuclei at the transition zone, “bowl of spaghetti” nuclei in the pachytene zone, and condensed chromosomes at diakinesis [37•]. By contrast, proliferating germline stem cells are absent in adult Strongyloididae. In female *Strongyloides ratti*, the entire distal arm is filled with giant nuclei, followed by a band of compact small nuclei at the gonadal loop. The germline organization in male *S. ratti* is basically similar to the female one except strongly condensed small nuclei at the end of the gonad [37•]. Staining based on histone modification showed similar staining patterns in male and female *S. papillosus*, *S. ratti*, and *Parastrongyloides trichosuri* (*P trichosuri*) [37•]. Parasitic female *S. ratti* and *S. papillosus* and free-living males showed strikingly similar patterns staining with H3K4me3 and H3Pser10 regardless of the absence of meiosis in parasitic females [37•]. The distal gonads of free-living female and male *S. ratti* and *S. stercoralis* have syncytial zones that may be used as microinjection sites for recombinant DNA to generate transgenic parasites [38•].

Most *Strongyloides* spp. and *P trichosuri* follow XX/XO sex determination mechanisms suggesting that XX/XO sex determination is ancestral in the family Strongyloididae [39]. In *Strongyloides* spp., meiotic recombination only occurs in the free-living generations because females of the parasitic generation reproduce by mitotic parthenogenesis [29]. Due to the presence of an extra X chromosome, parasitic females are capable of producing male and female offspring parthenogenetically. The molecular, cellular, and genetic mechanisms that control parthenogenesis in *Strongyloides* spp. are poorly understood. In *S. ratti* and *S. stercoralis*, both sexes have two pairs of autosomes, and the females have two X chromosomes (2*n* = 6) but the males have only one (2*n* = 5) [40]. Linkage mapping in *S. ratti* showed the presence of recombination in all three of its chromosome pairs, with a much lower frequency of these events in X chromosomes [41].

In *S. papillosus* and *S. vituli*, fusion of chromosome I and the X chromosome result in formation of a hemizygous region [42]. Females carry two pairs of a large (L) and a medium (M) (2*n* = 4) chromosomes. Males undergo sex-specific chromatin diminution where the middle of one L chromosome is removed by programmed DNA elimination to form two fragments, which are retained as separate chromosomes, with the result that 2*n* = 5 chromosomes [29]. The region undergoing chromatin diminution is surrounded by noneliminated chromatin and two breakpoints that occur between the eliminated and noneliminated region of one of the large chromosome [29]. Comparative miRNA analyses of different developmental stages of *S. ratti* and *S. papillosus* revealed similar results suggesting that miRNA has no role in sex-specific chromatin diminution in *S. papillosus* [43]. We know very little about how chromatin diminution happens and its mechanism of inheritance in *Strongyloides* spp. In *S. papillosus*, males transfer the intact large chromosome but not the diminished chromosome to their offspring. However, the females randomly pick and pass one of the two large chromosomes [42]. Genetic recombination in *S. papillosus* (2*n* = 4) occurs only in nondiminished chromosomes in both sexes [42]. The diminished regions of the *S. papillosus* chromosome are homologous to the X chromosome in *S. ratti* [42, 44]. We know little about the discrepant patterns of the inheritance of the sex chromosomes in these two species. The diminished region in the *S. papillosus* chromosome carries a large number of genes with known biological functions in *C. elegans* [42]. In addition to chromosomal differences, environmental factors such as the host immune status also affect sex determination in *Strongyloides* spp. In the case of *S. ratti*, a strong immune response against it in the rodent host results in a higher proportion of males in the fecal culture [45].

### Host Specificity, Genetic Diversity, and Zoonoses

The genus *Strongyloides* contains about 50 species which are all obligate gastrointestinal parasites of various vertebrates including humans, birds, amphibians, reptiles, and several mammals [13]. *Strongyloides* species are generally host-specific, but the medically important human parasites, *S. stercoralis* and *S. fuelleborni*, are known to have broad host range [3, 15••, 46]. Natural *S. stercoralis* infections have been identified in humans, dogs, cats, and several nonhuman primates [8–11, 18, 21, 47–49]. Recently, molecular and genetic studies in *S. stercoralis* isolated from different hosts and locations revealed the presence of huge genetic variations between these isolates. Jaleta et al. [15••] isolated *S. stercoralis* from humans and dogs in the same household in rural Cambodia and conducted single worm molecular genotyping using the nuclear 18S rDNA hypervariable region (HVR) I, HVR-IV, the mitochondrial *cox1* gene and single worm whole genome sequencing. HVR-I is generally used to detect polymorphisms within the same species *Strongyloides* spp., whereas HVR-IV is invariable within the same species and therefore usually used for species identification. The maternally inherited mitochondrial *cox1* gene is generally used to identify distinct haplotypes and cryptic species in *Strongyloides* molecular taxonomy. Genotyping of more than 500 single worms using HVR-I as a marker revealed more than 5 genotypes with T/A substitutions at position 458 and a stretch of 5T/4T consisting of a single base indel at position 176–179. A genotyping scheme using HVR-IV revealed two genetically distinct populations of *S. stercoralis* in these two hosts. One population occurred only in dogs, while the other population was present in dogs and humans. The phylogenetic relationships inferred using both nuclear 18S rDNA HVR-IV and mitochondrial *cox1* sequences were basically similar suggesting strong separation of human–dog and dog-specific strains of *S. stercoralis*. Nagayasu et al. [17] also reported the existence of two genetically distinct lineages and clades of *S. stercoralis* isolated from dogs and humans in Japan and Myanmar using a genotyping scheme similar to that of Jaleta et al. [15••]. Several *S. stercoralis* larvae with different HVR-I and IV genotypes were identified from the same dogs, and there was no evidence of crossing among larvae with different genotypes [15••]. These results provided the first direct evidence of zoonotic and canine-specific populations of *S. stercoralis*, which would constitute cryptic species of *S. stercoralis* present only in domestic dogs and not in humans [15••, 16, 17].

*S. fuelleborni* infections have also been widely reported in humans and several nonhuman primates such as chimpanzees, gorillas, macaques, and baboons [3–5, 12]. However, due to the absence of autoinfection in *S. fuelleborni* infections, the disease associated with them in humans is not as severe as in *S. stercoralis* infections [46]. The single worm genotyping scheme for *S. stercoralis* described above has also been widely applied to *S. fuelleborni* isolated from humans, nonhuman primate hosts, and different geographic locations. These studies revealed large genetic variations and several distinct clades of *S. fuelleborni* depending on the host and geographic locations where the parasites were collected [4, 5]. HVR-IV and *cox1* genotyping of *Strongyloides* spp. isolated from stools of humans, gorillas, and chimpanzees in Central African Republic and Uganda identified several distinct sequence types of *S. fuelleborni* [4]. Distinct haplotypes of *S. fuelleborni* from humans and long-tailed macaques in Thailand and Laos were also described and phylogenetically characterized using partial *cox1* sequences [5]. Three distinct clades of *S. fuelleborni* and other cryptic *Strongyloides* spp., which were close to *S. stercoralis* isolated from primates, were also discovered in Malaysia using partial *cox1* sequence genotyping [3].

*S. stercoralis* and *S. fuelleborni* are the two species of *Strongyloides* with zoonotic potential and so pose a serious public health risk in endemic areas. The presence of broad genetic diversity in *S. stercoralis* and *S. fuelleborni* indicates a complex genetic mechanism by which these parasites adapted to human, nonhuman primate, and canine hosts and to diverse geographic locations. To minimize the public health risks of *S. stercoralis* and *S. fuelleborni*, strongyloidiasis control and prevention strategies should include regular mass anthelmintic treatment of dogs and captive and semidomestic primates. However, to design and formulate strong and effective control and prevention strategies, several comprehensive and large-scale comparative genotyping and population genetic studies should be conducted in potential reservoir hosts and geographical regions in endemic areas. Recent molecular genetic studies focusing on the zoonotic potential of *S. stercoralis* and *S. fuelleborni* isolated from humans, dogs, cats, and nonhuman primates are presented in Table 1.

*S. papillosus* is generally known to parasitize sheep and several other domestic and wild ruminants [13]. However, Eberhardt et al. [52] found *S. vituli*, which differs genetically from *S. papillosus*, to be the predominant member of the genus-infecting cattle. It could be possible that most domestic and wild ruminants are infected with genetically diverse *S. papillosus* and other distinct *Strongyloides* spp. *S. papillosus* can cause fatal illness in lambs, kids, and calves kept under intensive management. Regardless of its significant veterinary importance, there are very few studies reported to describe the genetic diversities and population genetics of this group of animal parasites.

### Physicochemical Communication Between *S. stercoralis* and Its Host

*Strongyloides* spp. infect their mammalian hosts by active penetration of skin in a manner similar to the percutaneous route of infection by larval hookworms [31]. Infective iL3 develop in the soil either directly from L1 voided in the feces or indirectly via a generation of free-living males and females. Castelletto et al. [53] aptly characterized iL3 of *Strongyloides* spp. as fast migrating “cruisers” that disperse away from feces, close upon a stationary host and exhibit stereotypic behaviors such as nictation that enhance the likelihood contacting it. Recent reviews [54, 55] effectively capsulize findings to date on mediation of host seeking behaviors by physical and chemical cues emanating from the host. Briefly, host seeking by environmental iL3 of parasitic nematodes comprises orientation along gradients of heat and both volatile and soluble chemicals from the host. Within physiological limits, *S. stercoralis* migrates up a temperature gradient approximating the transition from the environment to the host skin and body temperatures. However, temperatures experienced by *S. stercoralis* iL3 in the hours leading up to a host encounter may significantly affect the proportion of larvae that migrate up the gradient [54, 56•] with lower environmental temperatures increasing the likelihood of iL3 migrating toward host body temperature. Temperature may also determine responsiveness of *S. stercoralis* larvae to chemoattractants [57].

Soluble attractants include components of sweat such as sodium chloride, serum factors, and a mammalian skin constituent, urocanic acid [55, 58–60]. Among potential volatile mediators, CO_2_ repels *S. stercoralis* iL3 at high concentrations and is neutral at lower ones. Intriguingly, almost all of the host-emitted volatile compounds from a large panel *S. stercoralis* iL3 attractants also attract mosquitoes to mammalian hosts [53, 55].

The most significant recent advancement in studies of host seeking and other chemically mediated behaviors in soil-transmitted nematodes is marked by the advent of targeted mutagenesis by CRISPR/Cas9 in *Strongyloides* spp. [61••, 62] and its application to discerning function in molecular components of sensory neuronal signaling. CRISPR/Cas9 knockout (discussed in detail below) of *Ss*-TAX-*4*, which encodes one of two subunits of a cGMP-gated ion channel in sensory neurons, demonstrated that this gene is required for normal thermotaxis by *S. stercoralis* iL3 [54, 56•]. Given that TAX-*4* is highly conserved in sensory neurons of nematodes, it will be interesting to see whether knockouts of this gene also affect chemotaxes as well.

Once *S. stercoralis* iL3 have penetrated the host skin, they must initiate a lengthy somatic migration that ultimately takes them to the lung and then to the small intestine. The calcium binding protein venestatin, recently discovered in the hypodermis and intestinal epithelium of *S. venezuelensis* iL3 and in their excretory secretory products, appears to be required for migration of these infective larvae from the skin to the lungs of experimentally infected mice [63]. Immunization of mice against venestatin reduces burdens of larvae in the lungs and intestine on days 2 and 3 of a challenge infection with *S. venezuelensis* iL3 [63], at once indicating the requirement for this protein in normal migration of the parasite in its host and the potential of venestatin as a vaccine target against *Strongyloides* spp. and possibly other soil-transmitted helminths.

### Animal and In Vitro Models for the Study of Human Strongyloidiasis

Animal and in vitro models are essential for maintaining *Strongyloides* spp. in the laboratory and for experimentation to elucidate basic molecular mechanisms involved in the infectious process and to discover new drug and vaccine targets in these parasites. The Mongolian gerbil constituted the first small animal model of both uncomplicated and hyperinfective *S. stercoralis* infection [64]. Initial work by Nolan and colleagues [64] on the gerbil model involved an *S. stercoralis* strain of canine origin. A notable recent development is the experimental infection of gerbils with a human isolate of *S. stercoralis* [65]. This achievement underscores the utility of the gerbil model as a tool for long-term maintenance of field isolates of *S. stercoralis* and its applicability to the study of zoonotic transmission of this parasite between humans and dogs. The power of the gerbil model lies in the susceptibility of this rodent species to infection with *S. stercoralis* and the fidelity with which it recapitulates the infective and autoinfective cycles of this parasite. While the gerbil model is invaluable for parasitological study of *Strongyloides* spp., the lack of highly inbred strains and of the ability to engineer its genome and the paucity of antibody and cytokine reagents limit the gerbil’s value as a subject for immunological study compared to murine models of parasitic nematode infection. Previous attempts to establish patent infection with *S. stercoralis* in immunocompromised mice have met with limited success, so the recent discovery that the NOD/LtSz-*scid* IL2Rγ^*null*^ (NSG) mouse can support both uncomplicated and hyperinfective *S. stercoralis* infection is highly significant [66••]. The capacity to engraft the NSG mouse with cytokine-mobilized stem cells that give rise to multiple components of the human immune system [67] opens broad avenues for in vivo research in human immunology and in immunoparasitology. However, neither the gerbil nor the mouse is a natural host of any *Strongyloides* species, and generally, inocula exceeding 100 iL3 are required to establish a patent infection with *S. stercoralis* in these animals. While this level of susceptibility is sufficient for routine strain maintenance, it represents a significant hurdle to establishing stable lines of genetically defined or transgenic *S. stercoralis*, where numbers of founding iL3 are frequently limited to 10 or 20 [61••, 68]. One remedy for this problem has been to utilize species of *Strongyloides* that naturally parasitize rats, *S. ratti* and *S. venezuelensis*, as subjects for the establishment of such lines. In their excellent review [69], Viney and Kikuchi discuss many aspects of the biology of these rat parasites with particular emphasis on their utility in investigations of host immunity and its effects on parasite biology and in the derivation of genetically defined parasite lines. In the case of *S. ratti*, the fact of a well-adapted pairing of parasite and rat host allows for the establishment of patent infections with a single inoculated iL3 in most attempts. This property has allowed the establishment of stable transgenic lines of *S. ratti* [38], and it promises to facilitate establishment of lines carrying mutations induced by CRISPR/Cas9 [61••]. It is noteworthy that once stabilized in rats, lines of transgenic *S. ratti* can be maintained for several months in gerbils (Lok et al., unpublished).

Advances in in vitro culture technique for both parasites and host tissues will also enhance laboratory models for the study of strongyloidiasis. Agar plate culture is essential for phenotyping the free-living stages of parasitic nematodes. Initial approaches to agar plate culture have involved direct application of conditions originally derived for the free-living nematode *C. elegans*. These comprise plates containing nematode growth medium (NGM) agar with lawns of the OP50 strain of *Escherichia coli* bacteria [70]. While this system supports survival of *Strongyloides* spp. including *S. stercoralis* and *S. ratti*, the fecundity and longevity of the free-living stages of *Strongyloides* spp. reared on such plates is significantly compromised. In response, Dulovic and co-workers [71] undertook a systematic effort to optimize agar plate culture conditions for *S. ratti* and arrived at a combination of V12 agar with a lawn of the HB101 strain of *E. coli* that optimizes brood size, egg hatchability, and adult longevity in cultured free-living stages of this parasite. This and similar advances in culture technique for *S. ratti* and other *Strongyloides* spp. stand to facilitate derivation of transgenic and other genetically defined lines of these parasites, which has been hampered in the past by suboptimal culture systems.

The first encounter between *Strongyloides* spp. and their mammalian hosts involves contact and penetration of host skin by iL3. Studies deploying in vitro models of skin penetration by iL3 of *Strongyloides* spp. and hookworms such as *Ancylostoma* spp., which may also invade the host percutaneously, have revealed that this event may trigger early events comprising reactivation of these developmentally quiescent infective larvae [72, 73]. These model systems generally involve a glass apparatus, dubbed a PERL chamber that comprises upper and lower compartments filled with a physiological medium, the lower compartment being warmed to host body temperature, and separated by a layer of mammalian skin recovered from some laboratory animal at necropsy. Percutaneous migration of iL3 in such an apparatus stimulates early markers of reactivation such as resumption of feeding in the hookworm *Ancylostoma caninum* [73] and upregulation of an aspartic protease (and vaccine candidate) encoded by *Spa-asp-2* in *S. papillosus* [72]. Skin samples used in such PERL chambers can constitute a source of technical variation due to their species of origin, thickness, inclusion of subcutaneous fat, storage parameters (frozen or fresh), and other factors that are difficult to control. In view of this, the achievement of an engineered three-dimensional skin equivalent culture, derived entirely from continuous lines of component cells [74], represents a very significant advancement in the study of percutaneously invasive larvae of parasitic helminths. In this instance, *S. ratti* iL3 were unable to penetrate the full-thickness skin equivalent structures but could traverse a cell-free collagen scaffold. By contrast, cercariae of *Schistosoma mansoni* could penetrate the full-thickness skin equivalents. Therefore, while technical modifications are required before this system can be deployed for studies of parasitic nematodes, engineered full-thickness skin equivalents seem poised to obviate the need for skin explants from euthanized animals and to eliminate the confounding variance inherent in using skin samples from a variety of sources and subjected to nonstandardized storage conditions.

### Manipulation of Cellular Signaling Pathways in the Infective and Autoinfective Processes

For over two decades, the morphological, behavioral, and molecular genetic aspects of dauer larval development in *C. elegans* have been regarded as a paradigm for framing hypothesis about the molecular signaling mechanisms that govern the development of iL3 of parasitic nematodes before, during, and after the infective process [75]. Published findings support that the canonical dauer regulatory signaling pathways in *C. elegans*, G protein-coupled sensory signaling, insulin-like signaling and steroid-nuclear receptor (NR) signaling involving homologs of the DAF-12 NR, and its dafachronic acid ligands are conserved in several soil-transmitted parasitic nematode species, including *Strongyloides* spp., and operate in sequence to regulate developmental arrest of iL3 in the environment and their reactivation following host infection [76]. The DAF-12 NR is conserved in *S. stercoralis* and *Ancylostoma caninum*, and developing larvae of these parasites respond to exogenous dafachronic acids in a manner consistent with dauer hypothesis [77]. That is, exogenous dafachronic acid stimulates resumption of feeding by iL3 of *Ancylostoma caninum* and *S. stercoralis* [77–79], just as it promotes the analogous process of dauer recovery in *C. elegans* [80]. Conversely, when applied to developing larval progeny of free-living *S. stercoralis* and *S. ratti* adults, dafachronic acid suppresses morphogenesis and developmental arrest of iL3 and promotes formation of second-generation rhabditiform fourth-stage larvae and reproductive adults, respectively [77, 78, 81]. Similarly, exogenous dafachronic acid can promote switching from the parasitic to the free-living developmental alternative in first-stage larval progeny of parasitic female *S. stercoralis* [78]. The studies mentioned above stressed phenotypic observations of parasitic nematode larvae following treatment with exogenous dafachronic acid and so left open the question of whether endogenous production of dafachronic acid ligands of known DAF-12 homologs occurs within the worms. Given that the final step in biosynthesis of dafachronic acids from dietary cholesterol in *C. elegans* is catalyzed by the DAF-9 cytochrome P450 (CYP), this question was addressed by assessing phenotypes resulting from blocking overall CYP function in *S. stercoralis* with the inhibitor ketoconazole. Ketoconazole treatment resulted in a dose-dependent suppression of resumed feeding by *S. stercoralis* iL3 under host-like culture conditions, and this phenotype could be rescued by exogenous Δ7-dafachronic acid, providing indirect evidence that endogenous dafachronic acid synthesis is required for this crucial event in the infectious process and indicating the potential of CYP function as a chemotherapeutic target in *S. stercoralis* and other soil-transmitted parasitic nematodes [78]. Further studies of DAF-12 signaling in nematodes revealed that in both *C. elegans* and *S. stercoralis*, this pathway also regulates genes involved in the aerobic catabolism of fatty acids, a process that supports reproductive growth of adult stages, both parasitic and free-living [82].

**Fig. 1:**
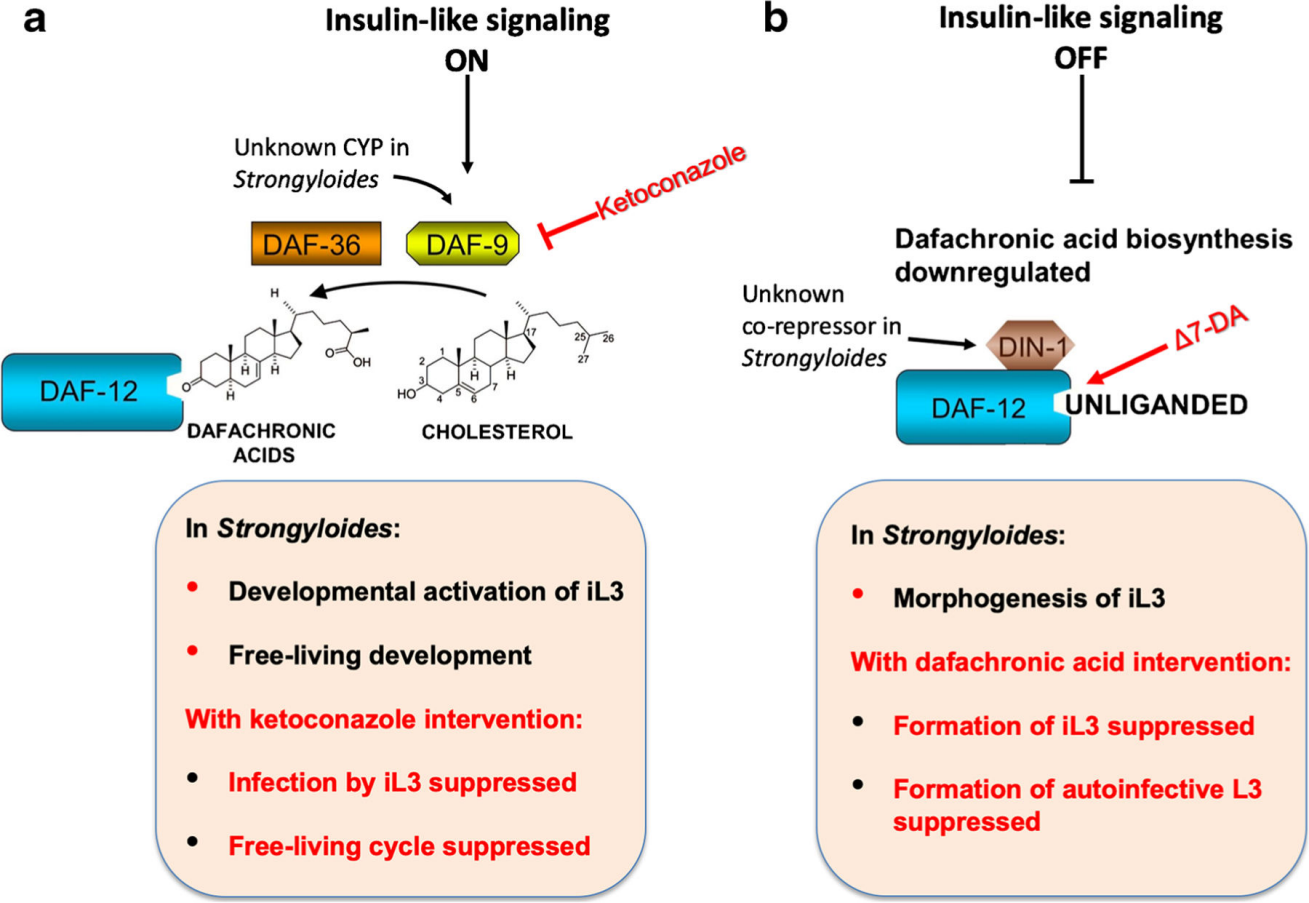
Administered small molecules can manipulate *Ss*-DAF-12 signaling to suppress infection and autoinfection by *S. stercoralis*. **a** Under natural conditions in nematode development, insulin-like signaling, along with TGFβ-like signaling, upregulates biosynthesis of dafachronic acids from dietary cholesterol. In *C. elegans*, the DAF-9 cytochrome P450 (CYP) catalyzes the final oxidative step in this biosynthetic pathway [80, 83]. DAF-12 signaling is conserved in parasitic nematodes, including *S. stercoralis* [77], and previous findings indicate that the cytochrome P450 inhibitor ketoconazole can block developmental activation of infective third-stage larvae (iL3) of *S. stercoralis* under host-like culture conditions and that this effect is partially reversed by the DAF-12 ligand Δ7-dafachronic acid [78]. This finding underscores the potential of CYP function in DAF-12 signaling as a chemotherapeutic target in blocking the infectious process by *S. stercoralis*. **b** Shutdown of insulin- and TGFβ-like signaling in *C. elegans* downregulates dafachronic acid biosynthesis, and in its unliganded state, DAF-12 downregulates dauer formation and upregulates continuous development [80, 83]. Likewise, in *Strongyloides* spp., administration of Δ7-dafachronic acid suppresses iL3 morphogenesis and promotes formation of second-generation free-living larvae and adults [77, 81]. Significantly, administering Δ7-dafachronic acid orally to NSG mice undergoing autoinfection with *S. stercoralis* also suppresses morphogenesis of autoinfective L3 [66••], underscoring the potential of *Ss*-DAF-12 signaling as a chemotherapeutic target in potentially fatal disseminated hyperinfection in human strongyloidiasis [84]

The ability of dafachronic acid to suppress the formation of iL3 in the post free-living generation of *S. stercoralis* [77, 78] prompted a hypothesis that when administered orally, this steroid might also prevent the formation of aL3 of this parasite within the guts of infected hosts and thereby prevent disseminated hyperinfection that causes the most serious complication of human strongyloidiasis. This hypothesis was supported by observations that 10 μM Δ7-dafachronic acid can suppress populations of *S. stercoralis* aL3 when administered in drinking water to infected NSG mice treated with methylprednisolone acetate to incite autoinfection [66••]. This finding is significant in that it constitutes evidence that DAF-12 signaling regulates the formation of aL3 in *S. stercoralis* and that this event requires downregulation of the DAF-12 ligand. This, along with the previous finding that the cytochrome P450 inhibitor ketoconazole suppresses developmental activation of *S. stercoralis* iL3 in a manner that can be partially reversed by Δ7-dafachronic acid, demonstrates how *Ss*-DAF-12 signaling can be manipulated by administered small molecules to either block the initial infective process (by ketoconazole or other cytochrome P450 inhibitor, Fig. 1a) or to suppress autoinfection (by Δ7-dafachronic acid or analog, Fig. 1b). This factor argues strongly for efforts to develop new chemotherapeutic agents targeting *Ss*-DAF-12 signaling to prevent infection, or, more practically, to suppress autoinfection in cases of in infected individuals. The latter intervention would serve to treat or prevent disseminated hyperinfection, the most serious, and potentially fatal, complication of human strongyloidiasis. Further discussion of the chemotherapeutic implications of this finding is discussed in a contemporaneous commentary [84].

*S. stercoralis* has also been used as a model in several recent studies of a family of RIO kinases that appear to be essential for normal development in this parasitic nematode and perhaps others [85–87]. RIO kinases constitute a family of atypical protein kinases that are named for the right open reading frame 1 domain that is a feature of the prototype for this family [85]. Of the three RIO kinases investigated in parasitic nematodes to date, RIOK-1 and RIOK-2 are conserved in taxa ranging from Archaea to humans [85, 87], while RIOK-3 is found only in multicellular eukaryotes [86]. RIOK-1 and RIOK-2 are both nonribosomal factors that are necessary for ribosomal biogenesis and normal cell cycle progression [85, 87]. The cellular function of RIOK-3 is less well understood but appears to diverge from those of RIOK-1 and RIOK-2. RIOK-3 is associated with 40s ribosomal particles, but an explicit function for it in ribosome biogenesis has not been demonstrated [86]. RIOK-3 is upregulated in cancer cells where it modulates NF-κB signaling and so is likely required for tumor growth [86].

Consistent with a function in ribosomal biogenesis in *S. stercoralis, Ss*-RIOK-1 is expressed in the cytoplasms of hypodermal cells and of neurons throughout the head, body, and tail of developing larval progeny of free-living adults. Phenotypes resulting from expression of a kinase-dead mutant *Ss*-RIOK-1 in post free-living larvae support that catalytic function of this protein kinase is required for normal motility and development of larval *S. stercoralis*, and indicate its potential as a chemotherapeutic target [87]. To our knowledge, developmental patterns of *Ss-riok-1* transcription have not been determined. *Ss*-RIOK-2-specific transcripts are most abundant in parasitic females and in larval stages adult females of the free-living generation. They are downregulated significantly in post free-living larvae and iL3 but are then significantly upregulated in host-activated iL3, dubbed L3+ [85]. In contrast to *Ss*-RIOK-1, *Ss*-RIOK-2 is expressed in the intestinal epithelium of post free-living larvae [85]. The divergent function of *Ss*-RIOK-3 compared to other members of the RIO kinase family is echoed by unique temporal and anatomical expression patterns in larval and adult *S. stercoralis*. Trends in abundance of *Ss*-RIOK-3-specific transcripts are like those of *Ss*-RIOK2 only in that they peak in parasitic females. Beyond that, *Ss*-RIOK-3-specific transcript levels are similar in post parasitic and post free-living larval stages with a slight decline in post free-living L1 [86]. Anatomical patterns of *Ss*-RIOK-3 expression shift from a one favoring expression in intestine and head neurons in post free-living L1 and L2 to one in which expression is limited to body wall muscle in post free-living iL3. Studies of RIO kinases in *S. stercoralis* drew upon the transgenesis platform that is available for this parasite. Details of this approach are discussed below in sections on manipulation of the genome and of gene expression in *Strongyloides* spp.

### The Genomes and Transcriptomes of *Strongyloides* spp.

With rapidly improving genomic and transcriptomic sequencing technologies, more efforts are being focused on assembly and complete annotation of the genomes of *Strongyloides* spp. of medical and veterinary importance to identify functional characterization of genes involved in development and parasitism. High-quality draft genome assemblies ranging from 42 to 60 Mb are currently available for *S. ratti* (43.1 Mb), *S. stercoralis* (42.6 Mb), *S. venezuelensis* (52.1 Mb), and *S. papillosus* (60.2 Mb) [88••]. *Strongyloides* genomes are small compared to the genomes of *C. elegans* (100 Mb) [89] and *P. pacificus* (169 Mb) [90]. Notably, *S. ratti* has the second most completely assembled and well-annotated nematode genome after that of *C. elegans*, with a high-quality 43 Mb reference genome comprising two autosomes and an X chromosome [88••]. The *S. papillosus* genome is assembled into approximately 4000 scaffolds making it the least well assembled and annotated of the *Strongyloides* spp. genomes. This is due to the level of chromatin diminution in *S. papillosus*, which hampers genome assembly and annotation [88••]. In the future, more effort should be devoted to improving the assembly and annotation of the genome of this important ruminant parasite. The *S. ratti* and *S. stercoralis* genomes have GC contents of only 21 and 22%, respectively, making them the most AT-rich nematode genomes reported to date [88••]. The total protein-coding contents of the *S. ratti and S. stercoralis* genomes range between 18 and 22 Mb, and the predicted numbers of genes for *S. ratti, S stercoralis, S. papillosus*, and *S. venezuelensis* are 12,451, 13,098, 18,457, and 16,904, respectively [88••].

Transcriptomic information about the gene expression profiles of different developmental stages have been reported for *S. ratti, S. stercoralis, S. venezuelensis*, and *S. papillosus* [44, 88••, 91]. In most of the comparative transcriptomic studies conducted to date, transcripts from astacin-like metalloproteinase, cysteine-rich secretory protein, antigen 5, and pathogenesis-related 1 protein (CAP) gene families were the most significantly upregulated in parasitic adult and iL3 stages [44, 88••, 91]. Notably, CAP and astacin gene families have undergone significant duplication and expansion in *Strongyloides* spp. [88••]. Apart from CAP and astacin gene families, genes encoding acetylcholinesterase, pyrolyl oligopeptidases, aspartic proteases, trypsin inhibitors, and transthyretin-like molecules are also greatly expanded, and their transcripts are highly abundant in parasitic adults and iL3 [44, 88••, 91, 92]. Products of these genes have been proposed as immunomodulatory molecules in parasitic nematodes [88••, 93], and their upregulation may be associated with infection of novel hosts, which makes members of these families primary candidates for genes associated with parasitism [30, 44, 88••, 91]. Hence, the biological functions of these proposed parasitism-related genes should be studied using molecular genetic tools to identify candidate genes for future treatment and control of strongyloidiasis. Unlike in *C. elegans*, there is lack of robust and effective functional genomic and genetic tools for most of the parasitic nematodes. However, the anatomical and morphological similarities of the *S. stercoralis* and *S. ratti* free-living females to *C. elegans* hermaphrodites have allowed researchers to adapt molecular genetic tools from *C. elegans* science to study basic biology and functional genomics in *Strongyloides* spp.

### The Proteomes

Gastrointestinal parasitic nematodes release numerous proteins into their surroundings within the host. These constitute the excretory and secretory proteins (the ES proteome), which modulate the immune systems of their vertebrate hosts, allowing the parasitic stages of the worms to survive longer [94]. Excretory and secretory proteins are likely crucial in establishing and maintaining parasitism by nematodes at all stages of infection, including recognition of the host, tissue penetration, somatic migration, and immune evasion throughout these processes [94–97]. These essential functions make proteins of the ES secretome promising targets for drugs or vaccines. The excretory and secretory proteins of *S. ratti* are well studied compared to other *Strongyloides* spp. Soblik et al. [97] identified 586 proteins in the *S. ratti* secretome. Hunt et al. [88••] identified 1266 proteins in the somatic proteomes of parasitic and free-living adult females of *S. ratti*. Of these, 569 are upregulated in parasitic adults and 409 proteins are upregulated in free-living adult females [88••, 92]. In addition, 882 ES proteins were also detected in this comparison, and 13% of the parasitic female ES proteins were products of genes upregulated in the transcriptome comparison [88••]. The presence of 25 astacin, 14 SCP/TAPs, prolyl endopeptidases, acetylcholinesterase, and transthyretin-like proteins in the ES proteome is consistent with upregulation of their encoding transcripts in the *S. ratti* and *S. stercoralis* parasitic females [88••]. A study of the secretome of *S. venezuelensis*, an obligate gastrointestinal parasite of rats, identified 436 proteins from iL3s and 196 proteins from parasitic females [98]. From those identified proteins, 350 were specific to iL3s and 106 were specific to the parasitic adult females, whereas 86 proteins were common to iL3s and parasitic adult females including proteins that contain astacin and CAP domains [98]. Most proteins secreted by *S. venezuelensis* iL3s are peptidases or are predicted to act in embryonic development and oxidation-reduction processes. Most ES proteins from parasitic female *S. venezuelensis* are associated with glycolysis or DNA binding [98].

Despite the efforts cited above, the functions and biological significance of most of the ES proteins from *S. ratti, S. stercoralis*, and *S. venezuelensis* remain largely unknown. Notable exceptions are two galectins, *Sr*-GAL-1 and *Sr*-GAL-3, excreted–secreted by *S. ratti* [96]. Galectins are a large group of β-galactoside-binding proteins which are involved in several biological processes especially in host–parasite interactions. The *S. ratti* galectins promoted release of the type 2 cytokines thymic stromal lymphopoietin and IL-22 from mucosal cells, supporting their immunoregulatory functions. They also stimulated cell migration in a dose-dependent manner [96]. Further studies to identify the biological functions of other salient ES proteins in parasitic females and iL3 stages of *Strongyloides* spp. would benefit the systematic search for anthelmintic drug targets.

### The Microbiomes

The gastrointestinal tracts of mammals are colonized by thousands of bacterial, viral, and fungal species that constitute the microbiota. The microbiota has important functions in gut physiology, metabolism, immunity, and maintenance of the gut epithelial barrier [99,100]. These beneficial effects largely depend on complex, mutualistic interactions between the gut microbiota, the host intestinal epithelium, and the host immune system [99, 101]. Parasitic helminths have adverse effects on the gastrointestinal tract of their hosts, altering of the gut ecosystem, including the composition of the microbiome, and epithelial barrier function through effects on mucus production and composition [101–103]. Helminths modify the host microbiome through the direct antimicrobial activity of their ES products [104]. Parasitic nematode infections can last for years owing to the ability of the worms to manipulate mammalian immune responses and thereby avoid immune-mediated expulsion [105]. Modulation of mammalian immune responses by helminths can also affect host immunity to concurrent infections resulting in impaired immunity to co-infection with various microbial pathogens in both humans [106] and in murine models [107, 108]. The presence of helminths in the gastrointestinal tract can alter both nutrient content and niche availability in this environment by impairing epithelial glucose absorption, which favors microbiota species that ferment sugars [109].

Like other helminths, *Strongyloides* spp. also exert immunomodulatory effects on their hosts [110, 111]. However, there have been relatively few studies to date of the effects of *Strongyloides* infection on the composition of the host microbiome. Comparison of fecal microbiomes from *S. stercoralis*-infected and noninfected human subjects in northern Italy revealed a significant increase in alpha diversities and a significant decrease in beta diversities in the infected individuals [112]. In addition, *S. stercoralis* infection was also associated with expansion and enrichment of *Leuconostocaceae*, *Ruminococcaceae*, *Paraprevotellaceae*, and *Peptococcus* and reduced *Pseudomonadales* populations compared to the samples from the uninfected subjects [112]. Longitudinal study of fecal microbiomes over the course of *S. venezuelensis* infection in mice [113] revealed that the abundances of several bacterial taxa in the host intestinal microbiome changed significantly as the infection progressed, with an increase in the genera *Bacteroides Candidatus* and *Arthromitus* and a decrease in the populations of *Prevotella* and *Rikenellaceae*. Notably, the compositions of the microbiota of *S. venezuelensis*-infected mice reverted to the preinfection state once the parasites were cleared from the host, suggesting that parasite-induced changes are reversible [113]. Further detailed comparative microbiome studies need to be conducted in *S. stercoralis* and *S. ratti* using rodent models in order to understand into the contributions of the host microbiome to the outcomes of *S. stercoralis* autoinfection, hyperinfection, and dissemination.

## Manipulating the Genome

### Transgenesis

The free-living generation of *Strongyloides* spp. provides access to the adult germlines of these parasites enabling gene transfer into germ cell nuclei by microinjection of transgene DNA into gonadal syncytia (reviewed in [62]) using methods developed for *C. elegans* [114–118]. Briefly, transgene-encoding plasmid vectors are expressed in tissue-specific patterns in F_1_ larval progeny of microinjected *S. stercoralis* and *S. ratti* [119–122]. Although robustly expressed in the F_1_ generation, these transgene sequences, which are presumed to incorporated into multicopy episomal arrays as they are in *C. elegans*, are silenced in subsequent generations [119]. However, integration of transgene sequences into the chromosomes of *Strongyloides* spp., either by means of a transposon such as *piggyBac* [68] or by CRISPR/Cas9 [61••], enables continuous transgene expression through sequential generations of host and culture passage and the establishment of stable transgenic lines of *S. ratti* [61••, 68]. It is noteworthy that CRISPR/Cas9 allows integration single-copy transgenes into precise genomic loci, constituting an advantage over transposon-based methods which, in the case of *piggyBac*, integrate many copies into random genomic loci, creating the possibility of confounding effects due to random insertional mutagenesis. Transient expression of transgene constructs in the F_1_ generation following gene transfer has been exploited to assess anatomical patterns of the expression of specific genes in *S. stercoralis*, including those encoding the RIO kinases of this parasite as discussed above [85–87].

The gonad of free-living *S. stercoralis* males also has a syncytial region, and microinjection of plasmid-based transgene vectors results in a transformation of a proportion of spermatocytes and maturing spermatozoa, which are transmitted to F_1_ progeny from crosses of microinjected males and nonmicroinjected females [38•]. Notably, crosses between free-living male *S. stercoralis* microinjected with a construct encoding a reporter transgene encoding green fluorescent protein and free-living females microinjected with a red fluorescent protein-encoding reporter yield a proportion of progeny expressing both reporters indicating contributions from both transduced parents [38•]. This discovery may provide the basis for generating homozygous mutations by CRISPRCas9, which is a newly developed capability in *Strongyloides* spp. discussed in the following section.

### CRISPR/Cas9

Arguably the most significant advancement in the 5 years that have elapsed since the last review of this topic in *Current Tropical Medicine Reports* is the demonstration of targeted mutagenesis in *S. stercoralis* and *S. ratti* by CRISPR/Cas9 [61••, 62]. Reliable systems for transgenesis in these parasites enabled the transfer of genes encoding the Cas9 endonuclease, specific and control guide RNAs, disrupting insert sequences and selectable fluorescent markers into the germlines of free-living female worms. This process created double-stranded breaks (DSB) in precise loci within target genes such as *Ss-unc-22* and *Ss-daf-16* and allowed DNA sequences containing stop codons and terminal arms homologous to sequences flanking the site of the DSB to be inserted into these loci by homology-directed repair [61••, 62]. Since all F_1_ progeny of free-living *S. stercoralis* and *S. ratti* adults must infect a host to continue their life cycles, mutant progeny cannot be propagated in culture. Consequently, mutant genotypes must be confirmed in individual *Strongyloides* larvae following phenotyping. The necessity for post hoc genotyping makes studies of putative CRISPR/Cas9 mutants in *Strongyloides* spp. more challenging and labor intensive than comparable studies in *C. elegans*, but the demonstrated feasibility of this technique nevertheless represents a major achievement in functional genomics for parasitic nematodes. In addition to initial reports of targeted mutagenesis in *Strongyloides* spp. using CRISPR/Cas9, the feasibility of this approach was demonstrated by Bryant et al. [56•] in using CRISPR/Cas9 mutagenesis to show that the neuronal cGMP-gated calcium channel TAX-4 is necessary to mediate thermotaxis in *S. stercoralis*.

## Manipulating Gene Expression

### RNA Interference

RNA interference (RNAi) is a biological process in which a double-stranded RNA molecule inhibits transcriptional and post-transcriptional gene expression [123, 124]. The natural functions of RNAi and its related processes are to protect the genome against the invasion of mobile genetic elements such as viruses and transposons [125]. RNAi has been harnessed as a tool to study the functions of genes in a wide range of organisms [126]. In experimental RNAi, double-stranded RNA (dsRNA) complementary to a portion of a gene of interest is administered to the subject via a variety of methods. dsRNA is processed into small interfering RNAs (siRNAs), which bind to the endogenous mRNA, forming a mRNA-siRNA duplexes and recruiting the RNA interfering specificity complex (RISC), which degrades/suppresses the target mRNA [126, 127]. The whole process results in the cessation/reduction of the effective function of the gene, which may result in informative phenotypes [126]. In nematodes, dsRNA/siRNA can be delivered by injection [128], soaking [129], feeding [130], and electroporation [131, 132]. The first successful use of RNAi in a gastrointestinal nematode parasite was reported in *Nippostrongylus brasiliensis*, a gastrointestinal parasite of the rat [133]. Subsequently, RNAi effects were reported in *Onchocerca volvulus* [134], *Haemonchus contortus* [131], *Ostertagia ostertagi* [135], *Ascaris suum* [136], and *Trichostrongylus colubriformis* [132]. In some cases, reduced target transcript levels were correlated with phenotypic changes in the subject parasitic nematodes. However, overall, RNAi effects on animal parasitic nematodes have been inconsistent, with variable results obtained between subject species, target genes, and experiments [137–140]. RNAi is apparently more efficient as an experimental tool in plant parasitic nematodes than in animal parasitic nematodes [138, 141].

Until recently, there have been no reports of informative RNAi effects in *Strongyloides* spp., regardless of the mode of delivery or configuration of the administered RNAi triggers. However, successful RNAi-mediated gene silencing was reported recently for *S. ratti* [142]. In this report, post parasitic *S. ratti* L1 were incubated at 19 °C for up to 4 days in RNAi culture medium which contains DMEM, octopamine (20 mM), *Sr*-DAF-*12* siRNA (10 mM), and RNAse out followed by phenotyping and quantification of *Sr-daf-12* transcripts by qRT-PCR. *Sr*-DAF-*12* knockdown significantly reduced the proportion of infective larvae arising from the homogonic or direct cycle from 12.67% in controls to 1.67% in worms undergoing *Sr-DAF-12*-specific RNAi. Free-living female *S. ratti* derived from *Sr*-DAF-*12* RNAi-exposed L1 produced fewer progeny than controls, and these grew more slowly and/or were less able to complete development to iL3 than controls [142]. Some larvae subjected to *Ss*-DAF-*12* knockdown developed to iL3 and were able to infect a susceptible host but exhibited low reproductive potential and shortened duration of infection compared to controls. Consistent with the regulatory function of *Ss*-DAF-*12* in *Strongyloides* fat metabolism [142], *Sr*-DAF-*12* RNAi knockdown also shifted aerobic fat metabolism toward anaerobic pathways. Previously, the application of exogenous Δ7-dafachronic acid to *S. papillosus* and *S. stercoralis* post free-living larvae prevented development to iL3s suggesting the importance of DAF-*12* and its ligand DA in the formation and metabolism of iL3s [66•, 77, 78, 81, 82]. Consistency and reproducibility have been significant issues in the application of experimental RNAi in animal parasitic nematodes [137–139]. It is very important to build upon the *Ss*-DAF-*12* study to optimize RNAi methods in *Strongyloides* spp. to obtain efficient knockdown and robust results to study the putative biological functions of parasitism genes, which could serve as candidate genes for future drug and vaccine targets.

### Expression of Dominant Interfering Transgenes

Assessing phenotypes produced by expression of dominant transgenes designed to either suppress or augment expression of their endogenous counterparts represents an alternative to transcriptional silencing of target genes by RNAi or their direct disruption or editing by CRISPR/Cas9 as methods of assessing gene function in parasitic nematodes. This approach was used to infer the requirement for the insulin-regulated transcription factor *Ss*-DAF-16 in the morphogenesis of the *S. stercoralis* iL3 [143] and has been used more recently to demonstrate the requirement for the catalytic activity of the RIO kinase *Ss*-RIOK-1 in development and survival of post free-living *S. stercoralis* larvae [87]. As previously discussed [144], both these studies involved transgenes designed to encode variants of their endogenous counterparts that retained the capacity to bind either genomic response elements in the case of *Ss*-DAF-16 or substrate in the case of *Ss*-RIOK-1 while having their functional domains ablated by mutation. It was assumed that plasmid-encoded transgenes are overexpressed in *S. stercoralis* by virtue of their being incorporated into multicopy episomal arrays. Thus, they outcompete the endogenous gene product of interest for binding partners but lack the capacity to execute their putative functions. In the case of *Ss*-RIOK-1, kinase activity of the dominant interfering transgene product was ablated by introducing a D282A mutation in sequence encoding its catalytic site; sequence encoding the substrate binding domain was left intact. Expressing this construct led to severe decrements in development and survivorship in worms expressing the dominant mutant construct [87], and these phenotypes were rescued with a frequency proportional to the expression level of a co-transformed transgene encoding wild-type *Ss*-RIOK-1.

### Molecular Diagnosis

Molecular diagnostic methods for human strongyloidiasis are based on the detection of parasite-specific DNA from stool, urine, and sputum samples using conventional PCR, nested PCR, and real-time PCR techniques [51, 145–147]. Usually, molecular diagnosis of *Strongyloides* spp. is done using the nuclear genetic markers such as the 18S ribosomal RNA gene (18S rDNA, also called SSU) or the mitochondrial marker, cytochrome *c* oxidase 1 (*cox1*). The 18S rRNA sequence is highly conserved in *Strongyloides* spp. and widely used for molecular taxonomy [148, 149]. Hasegawa et al. [150] reported several nucleotide polymorphisms among different species of *Strongyloides* in the four of the HVRs of the 18S rDNA. The nucleotide arrangements of a HVR-IV are species-specific and preferred as a molecular genetic marker for *Strongyloides* spp. identification and diagnosis [150]. In the HVR-I and the rest of the HVRs, some intraspecies variability has been reported in *S. stercoralis* and *S. fuelleborni* isolated from humans, dogs, and nonhuman primates [4, 46, 50]. Hasegawa et al. [46] also stressed the importance of the mitochondrial *cox1* gene to identify cryptic variations in *Strongyloides* spp. isolated from different geographic locations and host species.

Compared to the other diagnostic methods, molecular diagnostic techniques are highly specific. However, the sensitivity of these methods is greatly affected by the type and number of biological samples subjected to PCR and the length of the PCR fragments amplified and sequenced to identify parasite larvae in the samples. For example, each larval stage has a different quantity of DNA, and notably, eggs have very low DNA content. The *Strongyloides* cuticle may be very difficult to lyse, so optimizing DNA extraction techniques for each larval stage is also essential [15••, 16, 50]. In general, it is crucial to isolate worms from stool and other biological samples using an appropriate parasitological technique prior to DNA extraction in order to obtain DNA of sufficient quality and quantity for sensitive molecular diagnosis of *Strongyloides* spp. For definitive molecular identification and diagnosis, sequencing larger nuclear regions and whole mitochondrial genomes provides more relevant information than partial 18S rDNA and mitochondrial genetic markers. Sequencing whole genomes of individual worms would provide much more sequence and genetic information than using partial sequences of 18S rDNA and *cox1* for the molecular identification and diagnosis of *Strongyloides* spp. [15, 16].

## Conclusion

Strongyloidiasis, due primarily to *S. stercoralis*, affects more than 370 million people worldwide mainly in tropical and subtropical regions but is generally regarded as the most neglected soil-borne tropical disease. In the 5 years preceding this publication, groundbreaking accomplishments in genome sequencing and annotation, transcriptomics, and proteomics have facilitated the application of modern functional genomic methods such as transgenesis, CRISPR/Cas9, and RNAi to studies of *Strongyloides* spp. These new capabilities have already been brought to bear on functional studies of genes necessary for motor function such as *Ss-unc-22*, genes essential for cellular signal transduction such as *Ss-daf-12* and *Ss-daf-16*, genes involved in thermosensation such as *Ss-tax-4*, and the *Ss-riok* genes, which are associated with ribosomal genesis and function. Detailed studies of sex determination mechanisms and of germline organization in *Strongyloides* spp. have similarly uncovered unique features within this genus that may constitute specific adaptations to parasitism. Aside from their significant findings, these investigations underscore the power of *Strongyloides* spp. as models for the study of parasitic nematodes at the molecular and cellular levels. Well-annotated genomic resources have also contributed significantly to studies of the phylogeny and systematics of *Strongyloides* spp., uncovering evidence cryptic speciation within *S. stercoralis* sensu lato that has given rise to a canine-specific form and another one shared between dogs and humans that highlights the zoonotic potential of canine *Strongyloides* infections and calls for *S. stercoralis*-infected dogs to be treated as an adjunct to mass drug administration programs to control soil-transmitted helminths in humans. Finally, studies of steroid hormone signaling through the *Ss*-DAF-12 nuclear receptor have bolstered this pathway as a novel chemotherapeutic target in strongyloidiasis and perhaps other parasitic nematode infections. Among the many future research priorities in molecular and cellular biology and in ecology and epidemiology that are suggested by the accomplishments reviewed here are refining the CRISPR/Cas9 platform to allow precise sequence editing as well as gene disruption and to provide for host passage of worms carrying mutations in developmental regulatory genes and others affecting establishment in the mammalian host, better annotation of the *S. papillosus* genome, molecular surveillance to determine the prevalence and geographic distribution of zoonotic forms of *S. stercoralis* transmitted from dogs and other canids, and medicinal chemistry to improve drug-like characteristics of the dafachronic acid ligands of DAF-12 and promote their use as anthelmintic leads.

## Figures and Tables

**Table 1:** Summary of global molecular genotyping and single worm genome sequencing studies of *Strongyloides stercoralis* and *S. fueUehorni* isolated from humans, nonhuman primates, dogs, and cats

*Strongyloides* spp. identified	Host(s)	Country/location	Sample type	Molecular markers	No. worms genotyped	Reference
*S. stercoralis*	Human, dog	Cambodia	Stool	HVR-I, HVR-IV	290	[50]
*S. fuelleborni, S. stercoralis*	Human, chimpanzee, gorilla	CAR and Uganda	Stool	HVR-IV and *cox1*	29	[4]
*S. stercoralis*	Human, dog	Japan and Myanmar	Stool	Whole genome	57	[16]
*S. stercoralis*	Human, dog	Cambodia	Stool	HVR-I, HVR-IV, *cox1*, whole genome	766	[15••]
*S. stercoralis*	Human, dog	Japan, Thailand, Myanmar	Stool	HVR-I, HVR-IV, *cox1*, MSP gene	521	[17]
*S. stercoralis*	Human	Laos	Stool	18S rDNA, *cox1*	40	[51]
*S. stercoralis, S. fuelleborni*	Human	Thailand and Laos	Stool	18S rDNA, *cox1*	18	[5]
*S. fuelleborni*	Long-tailed macaques	Thailand and Laos	Stool	18S rDNA, cox1	96	[12]
*S. stercoralis*	Dog	Switzerland	Stool	HVR-I, HVR-IV, *cox1*	NA	[10]
*S. stercoralis, S. fuelleborni*	Orangutans, macaques, yellow and proboscis monkeys, slow loris	Malaysia	Stool	*cox1*	85	[3]
*S. stercoralis*	Cat	St. Kitts, West Indies	Colonic nodules	*cox1*	2	[9]
*S. stercoralis*	Dog	Spain	Stool	*18S rDNA*	12	[11]

*NA* number of worms sequenced was not stated in the article
